# Systemic high-dose intravenous methotrexate in patients with central nervous system metastatic breast cancer

**DOI:** 10.1186/s12885-019-6228-6

**Published:** 2019-11-01

**Authors:** F. Bazan, E. Dobi, B. Royer, E. Curtit, L. Mansi, N. Menneveau, M. J. Paillard, G. Meynard, C. Villanueva, X. Pivot, L. Chaigneau

**Affiliations:** 1 Department of Medical Oncology, University Hospital of Besancon, Besancon, France; 2 Department of Clinical Pharmacology and Toxicology, University Hospital of Besancon, Besancon, France; 3 Centre de Cancérologie du grand Montpellier, Montpellier, France; 4 Centre Paul Strauss, Porte de l’Hopital Strasbourg, Strasbourg, France; 5 Department of Medical Oncology, University Hospital Jean Minjoz, Boulevard Alexandre Fleming, F-25000 Besancon, France

**Keywords:** Central nervous system, Breast, Chemotherapy, Methotrexate

## Abstract

**Background:**

Infusion of high-dose intravenous methotrexate (MTX) has been demonstrating to penetrate the blood-brain barrier. The aim of this present study was to assess the efficacy and safety of high dose MTX in patients with central nervous system (CNS) metastases of breast cancer.

**Methods:**

Twenty-two patients with CNS metastases treated by MTX (3 g/m2) between April 2004 and October 2009 were enrolled. Clinical response rate, time to progression (TTP), overall survival (OS), and safety were assessed.

**Results:**

In terms of brain metastases, 2 patients (9%) achieved a partial response, 10 patients (45%) had disease stabilization, and 10 patients (45%) had disease progression. In others metastatic sites, 7 patients (39%) achieved a disease stabilization, and 11 patients (61%) had disease progression. TTP and OS were 2.1 (95%CI 1.4–2.9) and 6.3 (95%CI 1.8–10) months, respectively.

**Conclusion:**

High-dose MTX demonstrated a moderate activity at 3 g/m^2^. Nonetheless, the favorable toxicity profile should suggest the possibility to increase the dosage and further study are planned.

## Background

Central nervous system (CNS) metastases is a dismal evolution which results in devastating disease with progressive neurologic disability and death. Breast cancer is the second cause of CNS spread after lung neoplasia. Patients with metastatic breast cancer (MBC) are at high risk for CNS metastases with a rate of occurrence ranged between 10 to 42% [1–5]. Despite the progress made in treatment of extraneural metastatic breast cancer, therapeutic options for CNS metastases are limited and they provide a median survival shorter than 1 year [6]. Whole-brain Radiation Therapy (WBRT), is considered as a standard of care for brain metastases, and remains the most frequently used treatment. Rarely, small isolated unique (or in limited number) lesions are assessable to intend to cure strategy like surgery or « radio-gammaknife surgery » [7]. Systemic treatments provide a low rate of efficacy because most of chemotherapeutic agents are excluded from the CNS by the Blood-Brain Barrier (BBB) [8].

Among those agents, methotrexate (MTX) is active against breast and other primary cancers and is of interest by its ability to get through the BBB. Then, high-dose intravenous (IV) MTX reaches the CNS at an effective concentration able to control leptomeningeal metastases [9].

This open-label, single-institution, single arm, phase II trial, analyze prospectively the efficacy and safety of high dose IV MTX in patients with CNS metastases breast cancer.

## Methods

Twenty-two consecutive patients with CNS metastases of breast cancer were treated by high dose IV MTX at the university hospital of Besancon (Franche - Comte, France) between April 2004 and October 2009. This study was approved by the Institutional Review Board of the Regional Cancer Institute in March 2004. The cut off for data capture was May 31, 2011. Patients, disease and treatment characteristics, were included in the database. Metastases of the CNS included intra-cranial (cerebral and cerebellar), intramedullary and leptomeningeal metastases. CNS metastases were diagnosed by MRI and/or cerebrospinal fluid cytology. Patients were treated by IV MTX 3 g/m2 during 3 h infusion with concomitant hyperalkaline hydration. A rescue by IV folinic acid (40 mg every 6 h) was started 24 h after the completion of MTX until the blood concentration of MTX decreased below 0.05 μmol/l. MTX was administered every two or 3 weeks until the patient progressed clinically or radiographically. A pharmacokinetic assessment was performed to achieve a PK-PD study. Response rate, time to progression (TTP), overall survival (OS), and safety were assessed.

### Responses

CNS metastases and non-CNS metastases was assessed using Evaluation Criteria in Solid Tumors (RECIST 1.1) based on appropriate imaging every 12 weeks [10]. Taking into account that all patients were specifically assessed by neurologic exam and by brain MRI every 6 weeks. Leptomeningeal response was evaluated by cytologic examination every 6 weeks. For patients with both, parenchymal metastases and carcinomatous meningitis, progression in either site was assessed as progression of the disease. Patients with worsening neurological symptoms, regardless of the results of radiological controls or cytologic examinations, had a progressive disease. An objective response rate of 20% was determined as reasonable objectives for treatment with meaningful effect.

### Pharmacokinetics

Serum MTX concentrations were determined using a fluorescence polarization immunoassay (TDxFLx system; Abbott) following the manufacturer instructions at 24, 48 and 72 h after the end of the MTX infusion and later until the concentration was below 0.05 μmol/l.

The patients’ results were split in two groups with respect to their response assessment. The evaluation of the PK/PD relationship was performed using the concentration measured 48 h after the end of the MTX administration. An amount of 57 samples were analyzed. In each group the median number of concentrations assessed for each patient was 2 (range 1–4).

### Statistical analysis

OS is defined by the interval between the date of first treatment and date of death or last follow-up, using the Kaplan–Meier method. TTP is defined by the interval between time of first treatment and date of progressive disease or death, using the Kaplan–Meier method. Search for relationship between toxicities, efficacy criteria and pharmacokinetics parameters were performed by univariate and multivariate logistic regression. All statistical computations were performed by using software (SAS System for Windows, version 9.0, 2002; SAS Institute, Cary, NC), and results were declared significant at the two-sided 5% comparison wise significance level (*P* < .05).

## Results

### Patients characteristics (Table 1)

Twenty-two patients with MBC in CNS were treated by 67 cycles of high dose IV MTX (median 3, range 1–7). The median age was 59 years old (range, 37–84). Fourteen patients (64%) were treated for parenchymal disease, 5 patients (23%) for leptomeningeal metastases, and 3 (13%) for co-existent parenchymal and leptomeningeal disease. Twenty patients presented in addition to CNS lesions other distant metastatic disease.

**Table 1:** Patients characteristics

	N	%
Age Median: 59 y (37–84)		
Cycles of CT: 3 (1–7)		
BM	22	100
Parenchymal	17	77
Leptomeningeal	8	36
Lines prior of CT		
≤ 3	8	36
> 3	14	64
HER-2 status		
Positive	13	59
Negative	8	36
Not Known	1	5
Hormone Receptors status		
Positive	11	50
Negative	10	45
Not Known	1	5
Triple Negative	2	9
CNS Radiotherapy		
Yes	15	68
No	7	32
Other site of metastases		
Yes	20	81
No	2	9
Concomitant treatment		
Yes	14	64
Trastuzumab intravenous	11	50
MTX intrathecal	2	9
Lapatinib	1	5
No	7	32

Thirteen patients (59%) and 2 patients (9%) had respectively human epidermal growth factor receptor 2 (HER2) positive tumour and triple negative tumour.

Two thirds of patients received more than 3 lines of chemotherapy prior to high dose MTX. For the treatment of CNS metastases, 15 (68%) patients had received cerebral radiotherapy prior MTX treatment.

### Responses (Table 2)

**Fig. 1:**
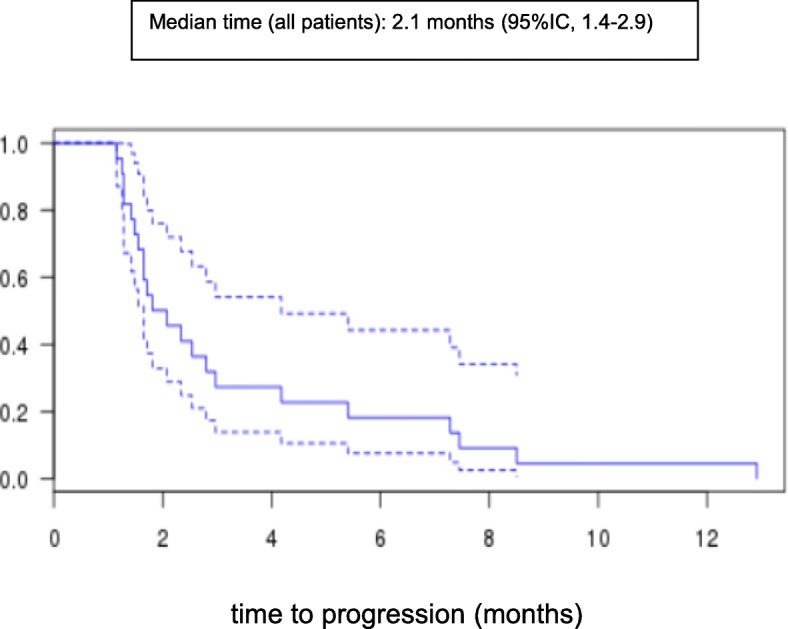
Kaplan-Meier plots of median time to progression (TTP) in all patients

**Fig. 2:**
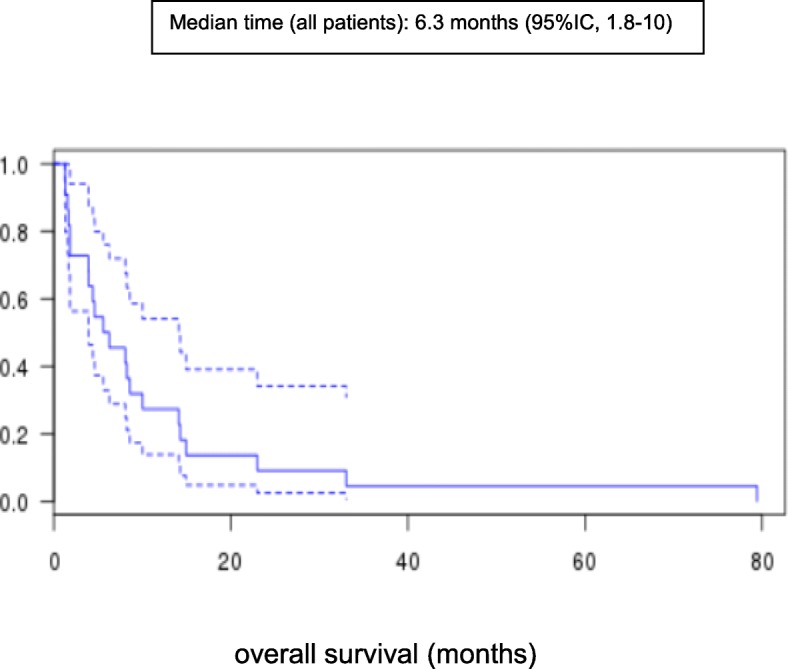
Kaplan-Meier plots of median overall survival (OS) in all patients

Twenty-two patients were assessed for CNS metastases and 18 patients for other metastatic sites. Regarding CNS evaluation, 2 patients (9%) achieved partial response, 10 (45%) had stable disease, and 10 (45%) had progressive disease. Both responding patients had meningeal disease. The responses were found to be partial; there was a loss of malignant cells in cerebrospinal fluid samples but incomplete regression of clinical and/or radiological abnormalities. The primary objective is not met, but 12 patients (55%) still have a disease control (objective response plus stable disease). Regarding the response in other metastatic sites, 7 patients (39%) achieved stable disease, and 11 (61%) had progressive disease (Table 2). The median follow up was 11 months. TTP and OS were 2.1 (95% CI 1.4–2.9) and 6.3 (95%CI 1.8–10) months, respectively (Figs. 1 and 2). After 11 months of follow-up, 21 patients had relapsed and 16 had died. A proportion of 27 and 10% of patients were alive at 1 year and at 2 years respectively.

**Table 2:** Responses

	Results	N	%
CNS *N* = 22	Complete Response	0	0
	Partial Response	2	9
	Parenchymal	0	0
	Leptomeningeal	2	9
	Stable Disease	10	45
	Parenchymal	7	32
	Leptomeningeal	1	5
	Parenchymal + leptomeningeal	2	9
	Disease control (partial response + stable disease)	12	55
	Progression Disease	10	45
	Parenchymal	7	32
	Leptomeningeal	1	5
	Parenchymal + leptomingeal	2	9
Other sites *N* = 18	Complete Response	0	0
	Partial Response	0	0
	Stable Disease	7	39
	Progression Disease	11	61

### Toxicity (Table 3)

All 67 cycles (mean 3, range 1–7) of high dose IV MTX were assessed for CTC v4. There was no grade 5 toxicity. Grade 3–4 haematological toxicities were observed in 4 patients (18%). Among them, one patient presented pancytopenia; the second one thrombocytopenia and neutropenia, and two others patients neutropenia or thrombocytopenia, respectively. One patient discontinued treatment after 7 cycles of because of serious grade 4 non-haematological toxicity presented as a « Lobster’s syndrome », characterized by seizure, hepatotoxicity and epidermal necrolysis. The most common grade 3 non-haematological toxicities were elevated serum hepatic transaminases and stomatitis in 4 (18%) and 2 (9%) patients, respectively.

**Table 3:** Clinical and Biological toxicity

Toxicity grade 3–4		N	%
Clinical	Stomatitis	2	9
Biological	Elevated serum hepatic transaminases	4	18
	Thombocytopenia	3	13.5
	Neutropenia	3	13.5
	Anemia	1	4.5

### Pharmacokinetics

A amount of 57 samples were analysed. In the non-responder group, the mean concentrations was 0.81 μmol/L (*n* = 24, range 0.03–5.64 μmol/L), whereas, for the responder group, the mean concentration was 0.59 μmol/L (*n* = 33, range 0.04–9.94 μmol/L). There was no significant difference with respect to the concentrations and toxicity when analyzing the data with a ANOVA with repeated measures.

## Discussion

Effective treatments for patients with CNS metastases are limited and there is an urgent need to found an active therapy. Radiotherapy and surgery are considered the gold-standard for brain metastases [2, 11–14]. For patient with leptomeningeal disease, palliative WBRT remains moderately efficient. Among additional options, the use of IT and IV drugs [15]. IV MTX is an alternative treatment when used at high dose because its capability to get though the BBB [8, 9]. Interestingly, in this present study, an objective response was observed in 9% and stable disease at 45% in the brain metastasis. The low rate of activity observed in the other distant lesions suggested an intrinsic tumour resistance to MTX. One could highlight the surprising discrepancies between the response rates in the brain versus outside. The result appeared at the opposite of what one could expect considering the well-established difficulties to access the brain versus other sites. The exposure to MTX was demonstrated to be equivalent in all sites, CNS included. A possible explanation, might be related to the lower exposure to prior chemotherapy in the brain lesions versus the other sites due to the BBB. This lower exposition induced less emergence in terms of mechanisms of chemotherapy resistance and could result in a more chemo-sensitive lesion.

In our study, there was not seen significant difference in relationship between toxicities, efficacy criteria and pharmacokinetics parameters with mean concentrations at 0.81 μmol/L and 0.59 μmol/ L for the non-responder and responder group, respectively.

Toxicity was acceptable and manageable in the present study using 3 g/m^2^ of MTX. The most common toxicity were reversible mild myelosuppression and elevated serum hepatic transaminases. Of note, one patient presented Lobster’s syndrome, characterised by seizure, myelosuppression, hepatotoxicity and epidermal necrolysis after 7 cycles of treatment.

Beyond traditional chemotherapy, targeted therapies and immunotherapies may provide a modest survival advantage [16–22]. Probably, improving the management of these patients will require a better understanding of the metastatic process through the BBB, the combination of the different therapeutic modalities available (surgery, radiotherapy, IT or IV chemotherapy, targeted therapy and immunotherapy), the exploration of new approaches and new molecules.

## Conclusion

High dose IV MTX is a possible option taking into account the pharmacokinetic and the brain exposure to the drug. Because a moderate activity was observed at 3 g/m^2^, without significant toxicities, one might consider that exploring higher dosage of MTX could be of interest. Based on this approach a phase I-II study aimed to assess higher dosage was undergoing.

## Data Availability

The datasets used and/or analysed during the current study are available from the corresponding author on reasonable request.

## Abbreviations

BBB: Blood-brain barrier
CNS: Central nervous system
HER2: Human epidermal growth factor receptor 2
IV: Intravenous
MBC: Metastatic breast cancer
MTX: Methotrexate
OS: Overall survival
TTP: Time to progression
WBRT: Whole-brain radiation therapy
